# Perceptions of Sex, Gender, and Puberty Suppression: A Qualitative Analysis of Transgender Youth

**DOI:** 10.1007/s10508-016-0764-9

**Published:** 2016-06-01

**Authors:** Lieke Josephina Jeanne Johanna Vrouenraets, A. Miranda Fredriks, Sabine E. Hannema, Peggy T. Cohen-Kettenis, Martine C. de Vries

**Affiliations:** 1 Department of Pediatric and Adolescent Psychiatry, Curium-Leiden University Medical Centre, Endegeesterstraatweg 27, 2342 AK Oegstgeest, The Netherlands; 2 Department of Pediatrics, Leiden University Medical Centre, Leiden, The Netherlands; 3 Department of Medical Psychology, VU University Medical Centre, Amsterdam, The Netherlands

**Keywords:** Gender dysphoria, Adolescents, Puberty suppression, GnRH

## Abstract

International guidelines recommend the use of Gonadotropin-Releasing Hormone (GnRH) agonists in adolescents with gender dysphoria (GD) to suppress puberty. Little is known about the way gender dysphoric adolescents themselves think about this early medical intervention. The purpose of the present study was (1) to explicate the considerations of gender dysphoric adolescents in the Netherlands concerning the use of puberty suppression; (2) to explore whether the considerations of gender dysphoric adolescents differ from those of professionals working in treatment teams, and if so in what sense. This was a qualitative study designed to identify considerations of gender dysphoric adolescents regarding early treatment. All 13 adolescents, except for one, were treated with puberty suppression; five adolescents were trans girls and eight were trans boys. Their ages ranged between 13 and 18 years, with an average age of 16 years and 11 months, and a median age of 17 years and 4 months. Subsequently, the considerations of the adolescents were compared with views of clinicians treating youth with GD. From the interviews with the gender dysphoric adolescents, three themes emerged: (1) the difficulty of determining what is an appropriate lower age limit for starting puberty suppression. Most adolescents found it difficult to define an appropriate age limit and saw it as a dilemma; (2) the lack of data on the long-term effects of puberty suppression. Most adolescents stated that the lack of long-term data did not and would not stop them from wanting puberty suppression; (3) the role of the social context, for which there were two subthemes: (a) increased media-attention, on television, and on the Internet; (b) an imposed stereotype. Some adolescents were positive about the role of the social context, but others raised doubts about it. Compared to clinicians, adolescents were often more cautious in their treatment views. It is important to give voice to gender dysphoric adolescents when discussing the use of puberty suppression in GD. Otherwise, professionals might act based on assumptions about adolescents’ opinions instead of their actual considerations. We encourage gathering more qualitative research data from gender dysphoric adolescents in other countries.

**Electronic supplementary material:**

The online version of this article (doi:10.1007/s10508-016-0764-9) contains supplementary material, which is available to authorized users.

## Introduction

Gender dysphoria (GD) is a condition in which individuals experience distress, because their gender identity (the psychological experience of oneself as male, female or otherwise) is incongruent with their gender assigned at birth (American Psychiatric Association, 2013). GD may exist in childhood, but only in a minority of prepubertal children will persist into adolescence. The percentage of “persisters” appears to be between 10 and 27 % (Drummond, Bradley, Peterson-Badali, & Zucker, 2008; Steensma, McGuire, Kreukels, Beekman, & Cohen-Kettenis, 2013; Wallien & Cohen-Kettenis, 2008). Treatment for prepubertal children consists of providing information, advice, psychological support, and/or family counseling. Those children who still experience GD when entering puberty, almost invariably will become gender dysphoric adults (de Vries, Steensma, Doreleijers, & Cohen-Kettenis, 2011). They may seek hormonal interventions such as puberty blockers (GnRH agonists) to suppress the development of secondary sex characteristics.

In recent years, the possibility of puberty suppression has generated a new but controversial dimension to the clinical management of adolescents with GD (Vrouenraets, Fredriks, Hannema, Cohen-Kettenis, & de Vries, 2015). The purpose of puberty suppression is to relieve suffering caused by the development of secondary sex characteristics, to provide time to make a balanced decision regarding the actual gender-affirming treatment (by means of cross-sex hormones and surgery), and to make passing in the new gender role easier (Cohen-Kettenis, Steensma, & de Vries, 2011). In the Netherlands, puberty suppression is part of the treatment protocol and as a rule possible in adolescents aged 12 years and older who are in or beyond the early stages of puberty and still suffer from persisting GD (Cohen-Kettenis et al., 2011). Occasionally, it is acceptable to start treatment at a (slightly) younger age than 12, if puberty has already started and is progressive. Earlier intervention might then make sense and, in fact, does already happen in practice.

An increasing number of gender clinics, including initially reluctant treatment teams, have adopted the Dutch strategy of puberty suppression (Vrouenraets et al., 2015), and international guidelines exist in which puberty suppression is recommended as a treatment option (Coleman et al., 2012; Hembree et al., 2009). Nevertheless, the use of puberty suppression is still controversial. Recently, we studied the opinions of 17 treatment teams worldwide. It was striking that the Standards of Care for GD of the WPATH and the guidelines for the endocrine treatment of individuals with GD of the Endocrine Society were considered too liberal by some teams, but at the same time too conservative by others (Vrouenraets et al., 2015). Many professionals working with gender dysphoric youth remain critical about the use of puberty suppression because of the lack of long-term physical and psychological outcomes (Korte et al., 2008; Viner, Brain, Carmichael, & Di Ceglie, 2005). Concerns have been raised about the risk of making the wrong treatment decisions, as gender identity could fluctuate during adolescence. Furthermore, adolescents might have poor decision-making abilities. Also, there may be adverse effects on health and on psychological and psychosexual functioning. Proponents of puberty suppression on the other hand emphasize the beneficial effects of puberty suppression on the adolescents’ mental health, quality of life, and of having a physical appearance that makes it possible to live unobtrusively in the affirmed gender role (Kreukels & Cohen-Kettenis, 2011). Several treatment teams, who work according to the guidelines, are exploring the possibility of lowering their current age limits for early medical treatment, even though they acknowledge the lack of long-term data (Vrouenraets et al., 2015).

In the literature on GD, it is mainly the professional view on the treatment that is available. Little is known about the way gender dysphoric adolescents themselves think about early medical intervention. However, to do justice to the developing autonomy of adolescents to make medical decisions, especially when it concerns far-reaching treatments, it seems appropriate to give serious consideration to the opinions of gender dysphoric youth themselves.

The aim of our project was to explicate the considerations and opinions of gender dysphoric adolescents in the Netherlands concerning the concept of sex and gender, and the use of puberty suppression in GD. Furthermore, we explored whether considerations and opinions on the use of puberty suppression of gender dysphoric youth themselves differ from those of professionals working in treatment teams, and if so in what sense. Therefore, we compared the results of the interviews with the adolescents to earlier data concerning the opinions of treatment teams worldwide (Vrouenraets et al., 2015). We paid extra attention to the perception of sex and gender in the media; the increased media-attention regarding transgender individuals as well as the imposed stereotype are discussed.

For this purpose, we have performed an empirical ethical study in order to answer the following questions: (1) What are the perceptions and views (direct thoughts or opinions) of gender dysphoric adolescents on puberty suppression in GD; (2) What are the (underlying) ideas, assumptions, and views of gender dysphoric adolescents about the concepts “best interests,” “autonomy,” and “sex/gender?”; (3) Do perceptions, views, and ideas on the use of puberty suppression of gender dysphoric youth in the Netherlands differ from those of professionals, and if so in what sense?

## Method

### Participants

The interviews were conducted in the context of a larger study on controversies surrounding puberty suppression in adolescents with GD. The study was approved by the institutional review board of the Leiden University Medical Center.

For the current part of the study, an empirical ethical approach was followed, using qualitative semi-structured interviews. Gender dysphoric adolescents were interviewed face-to-face in order to identify their considerations and opinions on the use of puberty suppression. The informants were 13 adolescents who were recruited from the Gender identity clinic in Leiden, the Netherlands. Fourteen consecutive adolescents, and their parents/guardians if the adolescent was younger than 18 years, were asked to participate when they attended their regular follow-up appointment. Thirteen adolescents and their families agreed to participate but one mother refused participation of her child. The adolescents who participated in the study were not selected in order to be representative, in characteristics (age, sex, socioeconomic status and psychopathology), of the population seen at the Curium-LUMC clinic. They were between 13 and 18 years of age, with an average age of 16 years and 11 months, and a median age of 17 years and 4 months. All adolescents, except for one, were treated with puberty suppression. The mean age at which the adolescents started treatment with puberty suppression was 15 years and 10 months. The adolescent who was not treated with puberty suppression immediately started treatment with cross-sex hormones because she was above the age of 18 when treatment was indicated, which is in line with the Dutch protocol. Five adolescents were trans girls (natal boys with a female gender identity) and eight were trans boys (natal girls with a male gender identity). The Full-Scale IQ of the interviewed adolescents ranged between 70 and 132, with an average Full-Scale IQ of 99 and a median of 102.

### Procedure and Measures

The interviewer was not involved in the diagnostics and treatment of these adolescents. The interviewer was a child and adolescent psychologist with a Master of Science degree and interview experience. Initial interview topics were formulated after examination of the relevant literature (see supplemental data). In accordance with qualitative research techniques, the interview topics evolved as the interviews progressed through an iterative process to ensure that the questions captured all relevant emerging themes (Britten, 1995; Guest, Brunce, & Johnson, 2006). The interviews contained general topics and no close-ended questions.

All interviews were audiotaped and transcribed verbatim. Before each interview informed consent for participation and tape recording was obtained from the interviewed adolescents as well as their parents in case the adolescents were younger than 18 years of age. The interviews with the adolescents took between 30 and 45 minutes. Data analysis was based on the constant comparative method (Malterud, 2001; Strauss & Corbin, 1998; Vrouenraets et al., 2015). We used an iterative process wherein we continually went back to the field and interviewed new participants to collect more data. The following processes of data gathering and analyses were used: (1) interviews; (2) transcription of the interview data; (3) open coding, which involved identifying relevant concepts in the text; (4) constant comparison of open codes, looking for conceptual similarities and differences; (5) identification of emerging themes; (6) continued sampling and interviewing as theoretical categories emerged and novel questions arose; and (7) continued coding and comparison of codes until nothing new was added to the theoretical categories. Data collection continued as long as new information came up. After no new content was found in the interviews, subject enrollment was stopped. This process, called thematic saturation, is a well-described qualitative method to avoid unnecessarily large and repetitive data sets (Guest et al., 2006).

The methodology and results of the interviews with professionals were previously published (Vrouenraets et al., 2015).

## Results

From the interviews with the gender dysphoric adolescents three themes emerged: (1) the difficulty of determining what is an appropriate lower age limit for starting puberty suppression; (2) the lack of data on the long-term effects of puberty suppression; (3) the role of the social context; this item consisted of two subthemes: (a) increased media-attention, on television and on the Internet, (b) an imposed stereotype. Representative quotations were chosen to illustrate the themes identified.

### The Difficulty of Determining What Is an Appropriate Lower Age Limit for Starting Puberty Suppression

The guidelines published by the WPATH and the Endocrine Society recommend the use of puberty suppression in adolescents when GD persists at the beginning of puberty (Coleman et al., 2012; Hembree et al., 2009). In principle, Dutch adolescents need to be 12 years of age and in pubertal Tanner stage 2–3 to be eligible for treatment with puberty suppression.

Most adolescents found it difficult to define an appropriate lower age limit. They saw it as a dilemma. On the one hand they thought it was important that children have the possibility of treatment with puberty suppression at the moment secondary sex characteristics of the natal sex start to develop, in order to prevent irreversible body changes like growth of breasts or breaking of the voice. This opinion is illustrated by the following quote:

I think it is hard to set an age requirement. On the one hand I think 12 years is a good age minimum, on the other hand I think that a transgender whose puberty started earlier should have the possibility to start treatment with puberty suppression before the age of 12. (trans girl; age: 13;11)

Another aspect that was mentioned was the issue of having enough time before making a decision regarding starting treatment with puberty suppression:

In my opinion 12 is a good age minimum because then these children and adolescents have time to consider what they want before making a decision regarding starting treatment with puberty suppression. (trans girl; age: 17;0)

The opinion to define different age limits for boys and girls because most boys mature later than girls do, was raised by another adolescent:

I would probably pick different ages for boys and girls. For girls I would put the age at I guess 11, and for boys I would put it at 13. Simply because biologically males mature later than females do. (trans boy; age: 18;5)

On the other hand, the adolescents have doubts about the competence of children to make decisions regarding topics like this:

Although a ten-year-old may realize what is going to happen in the short term, he or she might not be fully aware of the long-term consequences. (trans boy; age: 15;9)

### The Lack of Data on the Long-Term Effects of Puberty Suppression

There are limited data on the consequences of puberty suppression for bone mineral density (Klink, Caris, Heijboer, van Trotsenburg, & Rotteveel, 2015) and executive brain function (Staphorsius et al., 2015) but much remains unknown about the long-term effects of this treatment.

Most adolescents stated that the lack of long-term data did not and would not stop them from wanting puberty suppression. They said that being happy in life was more important for them than any possible negative long-term consequence of puberty suppression, as described by these three adolescents:

The possible long-term consequences are incomparable with the unhappy feeling that you have and will keep having if you don’t receive treatment with puberty suppression. (trans boy; age: 18;3)

I would rather live 10 years shorter but live a very happy life being myself, than live 10 years longer and be unhappy my whole life. (trans boy; age: 17;7)

It isn’t a choice, even though a lot of people think that. Well, actually it is a choice: living a happy life or living an unhappy life. (trans girl; age: 14;5)

Furthermore they mentioned that in order to be able to obtain long-term data, one person needs to be the first to undergo the treatment that needs evaluation, as these adolescents described:

If I were in charge I would definitely offer treatment with puberty suppression because that would make research feasible; people could sign up for studies in order to investigate possible consequences. (trans girl; age: 14;5)

The adolescents who were interviewed were more than willing to be that first person.

### Conceptualization of Sex and Gender in the Media

The third theme consists of two separate aspects.

#### Increased Media-Attention, on Television and on the Internet

The topic regarding the increased media-attention emerged during the interviews with the gender dysphoric adolescents and during the interviews with the professionals during a previous study (Vrouenraets et al., 2015). It was not mentioned before in the relevant literature.

In the last decade transgender individuals have become visible in the media. Many television programs, films, magazines, newspapers, and the Internet have paid attention to GD in children, adolescents, and adults. Recent examples are the documentary series in the Netherlands called “He is a she” featured in 2014, and again in 2016; the films The Danish Girl (2016), Boys Don’t Cry (1999), and Boys Meet Girl (2014); and the dozens of sites on the Internet that provide information on GD and transgenderism (Zucker, Bradley, Owen-Anderson, Kibblewhite, & Cantor, 2008). Some adolescents and professionals were positive about the increasing media-attention for transgender youth, others raised doubts about it. Some adolescents and professionals stated that this media-attention enables many transgender individuals to recognize their gender dysphoric feelings. They also stated that they had learned they were not the only ones having these feelings:

Thanks to media coverage I learned that gender dysphoria exists; that someone can have these feelings and that you can get treatment for it. … Beforehand I thought I was the only one like this. (trans boy; age: 18;11)

In my opinion the increasing media-attention is positive. I think that many transgender individuals only realize they are transgender after watching such television programs. (trans girl; age: 13;11)

Furthermore several adolescents stated that television shows and other programs have led to more acceptance in their social environment. Yet, some adolescents and professionals raised doubts about the increasing media-attention. Some adolescents mentioned that most transgender individuals in the media are people who are functioning quite well despite their gender dysphoric feelings. They believe however that this is not a representative picture of transgender individuals. For instance, the transgender individuals who also suffer from autism or depression are not shown. Furthermore, some adolescents thought that the media show a rather stereotypical picture of transgender individuals; as if all trans boys were tomboys from a very early age, did not like dolls and pink, but preferred playing with cars and playing soccer. They stated that not all transgender individuals are that “stereotypical”; and that some trans boys were even “girlish” when they were younger and preferred pink over blue. They wondered “why aren’t these transgender individuals in these television programs and in articles?,” as illustrated by the following two quotes:

Most of the trans men I have seen in the media … when they were younger, they always were stereotypical tomboys. I was personally quite stereotypically feminine; I liked drawing dresses with my mom, and still do. That made me feel alienated by the media. (trans boy; age: 18;5)

Television shows are never about girls that alternate between living like a girl and living like a boy. Such topics never appear. It seems such topics are simply not spectacular enough. (trans boy; age: 17;7)

#### A Binary Concept of Sex and Gender

Since July 2014 sterilization is no longer a requirement for transgender individuals in order to be able to change their gender on their birth certificate and on other official documents in the Netherlands (art. 1:28 subsection 1 DCC Jo art. 1:20 subsection 1 DCC). During the interviews most trans boys stated that they thought that they would not choose to have their ovaries/uterus removed because “it is not necessary anymore.” Before this change in law, many trans boys had their ovaries/uterus removed. A statement such as this one was typical among trans boys:

I don’t care about hysterectomy because changing gender on official documents is nowadays possible without this surgical procedure. (trans boy; age: 18;3)

The ability to change their gender on their official documents may be seen as imposing a binary phenotype on transgender individuals. Nevertheless, most adolescents thought that the concept gender is a continuum instead of a binary system, as this adolescent expressed:

In my opinion the concept gender is a continuum. In my case it is clear, I am a woman, but I know various transgender individuals who feel they are in between men and women. (trans girl; age: 13;11)

## Discussion

Using empirical methods this study aimed to explicate the considerations and opinions of gender dysphoric youth concerning the concepts of sex and gender, and the use of puberty suppression in GD. The interviews with the gender dysphoric adolescents were conducted in the context of a larger study on controversies surrounding puberty suppression in adolescents with GD. Besides the interviews with the adolescents, an extensive literature search was previously done as well as interviews with 36 professionals working in treatment teams worldwide (Vrouenraets et al., 2015). The data of the professionals enable us to compare their opinions with those of the adolescents.

Comparing the interviews of the adolescents with those of the professionals reveals that the adolescents and professionals do not agree about all topics. The lack of long-term data on possible side effects of the treatment for example was no problem for the adolescents, yet was a big issue for the professionals. In the interviews with the professionals, proponents remained cautious and opponents skeptical because of the fact that (long-term) risks and benefits of available treatments have not been fully established (Vrouenraets et al., 2015). One could explain the viewpoint of the adolescents by the fact that adolescence is a period in which short-term rewards are more important than long-term rewards, even when choosing for an immediate reward can mean a later loss or risk (Blakemore & Robbins, 2012; Crone & Dahl, 2012). However, the adolescents also showed that they seriously weighed the short- and long-term consequences, and consciously chose for the treatment. Furthermore, they showed a remarkable insight and altruism in their willingness to participate in research, which also meant they were able to look beyond their own short-term interests.

It is striking that several interviewed gender dysphoric adolescents gave arguments which were also mentioned by opponents among care providers, for example doubts about the ability of adolescents to make decisions regarding medical treatment at the age of 12 or younger. The adolescents sometimes seemed to be even more cautious than some of the professionals. Several of the interviewed professionals work in treatment teams that use the Dutch guidelines, but are exploring the possibility of lowering the current age limit for early medical treatment. However, defining an appropriate age limit appeared to be difficult for the adolescents. They questioned the competence to take complex decisions at a young age but also emphasize the importance of having the possibility to be treated with puberty suppression at the moment secondary sex characteristics of the natal sex develop. This made them feel setting an age limit as a dilemma.

A theme on which informants (both among professionals and adolescents) take diverging viewpoints regards the conceptualization of sex and gender in the media on television and on the Internet. Some adolescents and professionals think positively about the increasing media-attention, others raise doubts about it. Some speculated that information on television shows and on the Internet may have a negative effect and, for example, lead to medicalization of gender-variant behavior (Vrouenraets et al., 2015). Furthermore, according to some informants, the media do not seem to show a representative picture of transgender individuals. According to them most transgender individuals who are shown in the media function well despite their gender dysphoric feelings, even though that is not always the case in real life. Furthermore, the picture of transgender individuals that is conveyed to the public seems to be much less varied and complicated than the existing broad spectrum of transgender phenomena (Kuyper & Wijsen, 2014). This image depicts transgender individuals as much more binary than seen in real life; a complete transition is for example not the ultimate goal for all transgender individuals (Beek, Kreukels, Cohen-Kettenis, & Steensma, 2015). If it is true that a stereotypical and binary picture is mostly shown in the media, this might contribute to more acceptance of transgender individuals in society. However, this is at the expense of an understanding by the public of the full range of transgender phenomena.

After a change in the law, sterilization is no longer a requirement for transgender individuals to be able to change their gender in official documents in the Netherlands. Most interviewed trans boys thought that, after this change in the law, they would not choose to remove their ovaries and uterus because “it is not necessary anymore.” It should be noted that the requirement for transgender individuals to be able to change their legal gender is and was not the only reason for transgender individuals to remove their ovaries/uterus. Other reasons for desiring hysterectomy and/or oophorectomy are for example that transgender men may feel less female and more satisfied about their bodies after this type of surgery, may avoid problems with menstrual bleeding once puberty suppression is stopped or do not need to undergo investigations such as cervical smears to screen for cervical cancer. These reasons to undergo this type of surgery are discussed with the adolescents during the diagnostic and treatment phase. Yet, before the law change, many transgender individuals had their ovaries and uterus removed and may not have felt they had a true choice to undergo this surgery or not. Many may have felt forced to make the step in order to become their true gendered selves.

It is important to give voice to the gender dysphoric adolescents themselves, hearing their views on topics like gender, autonomy, and best interests, when discussing the use of puberty suppression in GD. As professionals publish their pleas for or against this treatment it only seems fair to add the views of those involved, the adolescents themselves, to the literature. This is important because otherwise professionals act upon assumptions on the adolescents’ views, rather than on the actual considerations and opinions of the adolescents. This is illustrated by the fact that the adolescents seem to have more concerns about lowering age limits than professionals. In order to advance the ethical debate, we need to continue discussing the various themes based on research data in addition to mere opinions. If not based on empirical data, ideas on GD treatment may diverge even more, which eventually may lead to (even more) inconsistencies between the approaches recommended by health care professionals across different centers.

There are strengths and weaknesses to the present study. The qualitative nature of the study made it possible to find out, in depth, the ways in which the adolescents think or feel about specific topics. Nevertheless, the considerations explicated in this study are solely from a relatively small sample of adolescents from the treatment team in Leiden, the Netherlands. The considerations of adolescents are likely to be different in other adolescents and in other countries or other clinics. Furthermore, all adolescents, except for one, started treatment with puberty suppression when they were older than 12, at an average age of 15 years and 10 months. This could have had an effect on the way these adolescents think about the current age limit of 12 years of age. These adolescents were never confronted with this limit which possibly did not make them feel the urge to lower the current age limit. It calls for studies among a larger group of adolescents who started treatment at younger ages and adolescents who were not treated with puberty suppression at all. Furthermore the adolescents in our study come from a society with a relatively high acceptance of transgender individuals (Keuzenkamp & Kuyper, 2013). Studies in other countries should be done, not only to investigate differences between youth that had different treatment regimens but also to investigate the relationship between their views and the culture they live in. Until more research data become available, the optimal timing of treatment in GD will remain unclear (Olson-Kennedy et al., 2016).

## Electronic supplementary material

Below is the link to the electronic supplementary material.

Supplementary material 1 (DOCX 496 kb)
