# Supplementary material for: Perceptions of Sex, Gender, and Puberty Suppression: A Qualitative Analysis of Transgender Youth
Source: Arch Sex Behav. 2016 Jun 1;45:1697–703. doi: 10.1007/s10508-016-0764-9 (PMC4987409; doi:10.1007/s10508-016-0764-9)
Supplement: Supplementary file 1 — Supplementary material 1 (DOCX 496 kb) [file 10508_2016_764_MOESM1_ESM.docx]

Supplemental data;

Initial Interview questions

First of all, could you describe how you came into contact with the genderteam in Leiden? Where did you hear about it? Which trajectory have you followed in order to start treatment with cross sex hormones?

I. How should the ‘best interest’ of a child be defined?

Questions to the adolescent:

1. Why do you feel it is important that adolescents with gender dysphoria should have the possibility to receive treatment with puberty suppression?

2. What do you feel should be the goal of the treatment with puberty suppression in adolescents with gender dysphoria?

II. What is the role of the (non-)availability of an explanatory model for gender dysphoria?

Questions to the adolescent:

3. Have you ever thought about a possible cause for gender dysphoria? What are your ideas in respect of the etiology of gender dysphoria?

III. Is gender dysphoria a (mental) illness or a social construct?

Questions to the adolescent:

At present, gender dysphoria is classified as a psychiatric disorder. Some people argue that gender dysphoria should not be classified as a mental illness, while others point out that the gender dysphoria diagnosis is important because of the need for diagnostic categories that facilitate access to healthcare, insurance companies and the communication between diverse professions.

4. How would you classify gender dysphoria, and why?

IV. Is gender correctly described as a dichotomy, or is the concept more fluid?

Questions to the adolescent:

Thinking about gender is often dichotomous (male-female). Transgender emergence however, does not necessarily mean a transition from male-to-female or female-to-male; for some adolescents (and adults) transition involves emergence as a bi-gender, pan-gender, or androgynous person. Trans-sexual people are not all just men trapped in women’s bodies and vice versa: the reality is more complex.

5. How would you describe the concept gender, as a dichotomy or as a more fluid concept, and why?

6. Do you already have thoughts about the kind of treatment (hormones and/or surgery) you would like to receive? Could you explain why you would (not) like to undergo certain kinds of surgery?

V. How to define ‘competence’ and ‘autonomy’ in a child with gender dysphoria?

Questions to the adolescent:

In the Netherlands, puberty suppression is part of the treatment protocol and is as a rule possible for adolescents aged 12 years and older who are in the early stages of puberty.

7. What is your opinion about the age minimum of 12 years?

8. From what age should children and adolescents have the possibility to receive treatment with puberty suppression according to you? Could you explain why?

9. Do you feel that all children/adolescents should have the possibility to receive treatment with puberty suppression from the age you just mentioned; or do you feel that other factors should be taken into account as well? (for example how long the gender dysphoric feelings are already present or how smart a child/adolescent is).

VI. Risks and benefits of treatment

Questions to the adolescent:

In the case of gender dysphoria (long term) risks and benefits of available treatments are not fully established. Some opponents feel it is not wise to give children/adolescents treatment with puberty suppression because they have concerns about the ability of children/adolescents to foresee the (possible) long-term effects.

10. What is your opinion about this topic?

Ending;

This interview is almost finished now. Are there any important topics you feel we forgot to discuss?

Emerging Interview questions

VII. Media-attention

Questions to the adolescent:

11. Have you ever watched a television program, a film that paid attention to gender dysphoric individuals, or have you ever read an article in a magazine, newspaper or on the Internet that paid attention to gender dysphoric individuals?

12. Some people are positive about the increasing media-attention for transgender individuals, others raise doubts about it. What are your ideas in respect of the (increased) media-attention for transgender individuals?
